# The association of three vaccination doses with reduced gastrointestinal symptoms after severe acute respiratory syndrome coronavirus 2 infections in patients with inflammatory bowel disease

**DOI:** 10.3389/fmed.2024.1377926

**Published:** 2024-03-18

**Authors:** Yu Hong, Tianyi Che, Xiangguo Shen, Jie Chen, Kui Wang, Lingying Zhao, Weitong Gao, Yao Zhang, Wensong Ge, Yubei Gu, Duowu Zou

**Affiliations:** ^1^Department of Gastroenterology, Ruijin Hospital, Shanghai Jiao Tong University School of Medicine, Shanghai, China; ^2^Department of Gastroenterology, Shanghai Wusong Central Hospital (Zhongshan Hospital Wusong Branch, Fudan University), Shanghai, China; ^3^School of Public Health and The Second Affiliated Hospital, Zhejiang University School of Medicine, Hangzhou, China; ^4^Department of Gastroenterology, Xinhua Hospital, Affiliated to Shanghai Jiao Tong University School of Medicine, Shanghai, China

**Keywords:** SARS-CoV-2 vaccine, three vaccination doses, gastrointestinal symptoms, inflammatory bowel disease, duration, activity

## Abstract

**Background:**

The protective efficacy of the severe acute respiratory syndrome coronavirus 2 (SARS-CoV-2) vaccination against the new-onset gastrointestinal (GI) symptoms following COVID-19 infection is critical among patients with inflammatory bowel disease (IBD); however, the optimal protective vaccine dose remains unknown. Therefore, this study aimed to clarify whether there is a correlation between SARS-CoV-2 vaccinations and GI symptoms following Omicron infection in patients with IBD.

**Methods:**

We conducted a multicenter cross-sectional study of IBD patients among three tertiary hospitals in eastern China. Professional physicians collected all data using online questionnaires. The patients were stratified into four groups: patients who were unvaccinated and patients who received one, two, or three vaccination doses. The primary outcome was the presence of any new-onset GI symptoms after SARS-CoV-2 infection before a negative SARS-CoV-2 nucleic acid test or a negative self-testing for antigens.

**Results:**

In total, 536 patients with IBD (175 unvaccinated, 31 vaccinated, 166 vaccinated with two doses, and 164 vaccinated with three doses) reported having COVID-19 infection. Compared with the unvaccinated, the three vaccination doses group was associated with reduced GI symptoms after infection (adjusted odds ratio = 0.56, 95% confidence interval 0.34–0.90, *P* < 0.05). Reduced diarrhea (adjusted odds ratio = 0.54, 95% confidence interval 0.31–0.92, *P* < 0.05) and nausea or vomiting (adjusted odds ratio = 0.45, 95% confidence interval 0.21–0.92, *P* < 0.05) were observed in the three vaccination doses group compared with the unvaccinated group.

**Conclusions:**

In conclusion, in the 536 patients with IBD who reported COVID-19 infection, we found that the three vaccination doses, but not the one or two doses group, were associated with reduced GI symptoms after infection compared with the unvaccinated group.

## 1 Introduction

Inflammatory bowel disease (IBD), which includes Crohn's disease (CD) and ulcerative colitis (UC), is a chronic immune-mediated inflammatory disease of unknown etiology. Research suggests that it results from the uncontrolled attack of the immune system on certain agents, which damages the gastrointestinal (GI) tract (1, 2). Viral infections of the GI tract initiate inflammatory events and may induce severe inflammatory disorders in patients with autoimmune diseases (3). SARS-CoV-2 infects the GI system (4), causes GI symptoms (5), and is associated with severe COVID-19 outcomes (6).

Disease activity can be influenced by behavioral factors, including mental stress, smoking, and medication adherence, which might have changed during the COVID-19 pandemic (7–9). During the pandemic, some patients with IBD experienced anxious-depressive symptoms (8) and reduced therapeutic adherence (9), triggering a disease flare. Consequently, new onset GI symptoms were observed following COVID infection in patients with IBD, including diarrhea, nausea or vomiting, anorexia, abdominal pain, and hematochezia (4, 5, 10), which linked patients with IBD to an increased risk of disease flare during the SARS-CoV-2 pandemic.

The SARS-CoV-2 vaccine exhibits a protective mechanism (11). Previous studies have shown the protective efficacy of vaccination in individuals with COVID-19 in preventing severe outcomes (12). Notably, several studies have focused on the efficacy (13), safety (14–16), and immunogenicity (17) of the SARS-CoV-2 vaccine in IBD patients. Using standardized disease activity scores (Mayo Partial Score and Harvey-Bradshaw Index), Pellegrino et al. showed that no adverse events or disease flares were associated with three doses of the COVID-19 vaccine in patients with IBD on biological therapy (18). The British Society of Gastroenterology's Inflammatory Bowel Disease suggests that patients with IBD should receive the SARS-CoV-2 vaccine and accept any approved SARS-CoV-2 vaccination offered to them (19).

SARS-CoV-2 has become more contagious, accompanied by various COVID-19 infections in the vaccinated population (12), many of whom were patients with IBD. With the recent adjustment of public health control measures in China, there were widespread SARS-CoV-2 Omicron infections in Shanghai during the autumn and winter of 2022 (20), and the disease activity of patients with IBD during the pandemic remains unknown. In addition, few studies have examined the efficacy of the inactivated COVID-19 vaccination in patients with IBD. Hence, we gathered self-reported patient data during the autumn and winter of 2022 from three tertiary hospitals in eastern China to investigate the potential correlation between SARS-CoV-2 vaccination and GI symptoms in COVID-19-infected IBD patients. This study aimed to investigate the impact of SARS-CoV-2 vaccines on GI manifestations in patients with IBD following COVID-19 infection and to furnish evidence concerning the vaccination decision for IBD patients.

## 2 Patients and methods

### 2.1 Participants and study design

We conducted a multicenter cross-sectional study between January and February 2023 in three tertiary hospitals in Shanghai, Eastern China. Notably, the attending physician collected all data from patients with IBD using an online tool, Wenjuanxing, the Chinese equivalent of SurveyMonkey. Supplementary material 1 provides a detailed online questionnaire.

**Figure 1:**
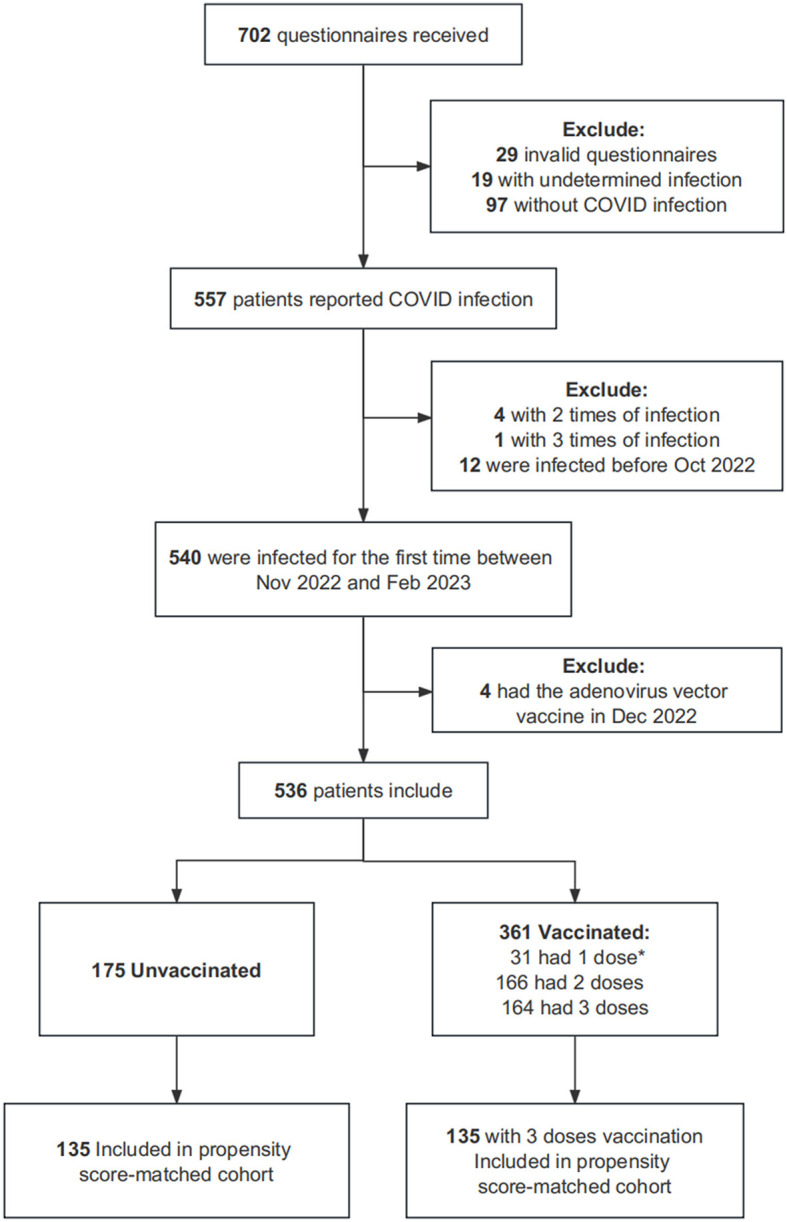
Inclusion criteria for a cross-sectional study showing the association between SARS-CoV-2 vaccination doses and GI symptomatic infection among patients with IBD. *Dose of SARS-CoV-2 vaccination.

The study included only participants with IBD aged ≥14 years, excluding children from the study group. IBD diagnosis was established through endoscopy, imaging, and clinical manifestations by at least two gastroenterologists from tertiary hospitals and was recorded in the electronic medical record system. In total, 1,500 patients with IBD were invited to participate in the study, and 702 questionnaires were received. We excluded 29 incomplete questionnaires, 116 participants without infection or with unconfirmed infections, 17 who were infected with COVID-19 more than twice or before October 2022, and 4 with the adenovirus vector vaccine in December 2022. Finally, 536 participants were included. Propensity scores were additionally calculated between the unvaccinated group and those who had received three doses of vaccination, and 270 participants were included in further sensitivity analyses (Figure 1). Supplementary Figure 1 summarizes the distribution of propensity scores. The exact COVID-19 infection dates of these patients were recorded between October 2022 and February 2023 (Table 1). According to the previous literature (20), the predominant sublineages of Omicron were BA.5.2 (68%), BQ.1 (12%), and BF.7 (11%) in the region's autumn/winter 2022 COVID-19 wave.

**Table 1:** The monthly onset of COVID-19 in 536 patients with IBD, stratified by vaccination status.

**Month**	**COVID onset cases (*N =* 536)**	**Unvaccinated (*N =* 175)**	**One dose (*N =* 31)**	**Two doses (*N =* 166)**	**Three doses (*N =* 164)**
October 2022	3	0	0	1	2
November 2022	10	2	1	4	3
December 2022	478	168	25	141	144
January 2023	53	6	5	23	19
February 2023	1	0	0	0	1

Supplementary Table 1 lists the detailed information on the 116 candidates who validated the questionnaire without confirming COVID-19. In total, 21 patients with confirmed infections did not meet our criteria listed in Supplementary Table 2. The ethics committee of Ruijin Hospital, Shanghai Jiao Tong University School of Medicine, approved the study protocol (ID: 2023-167). Informed consent was not required.

### 2.2 Exposure

This cross-sectional study assessed the efficacy of different vaccination doses for GI symptoms. The exposure of interest included IBD patients with one, two, or three doses of inactivated SARS-CoV-2 vaccination vs. those who were unvaccinated. The interval between vaccination and the onset of COVID-19 was at least more than 1 month (11). In December 2020, the SARS-CoV-2 vaccination was initiated for people with a high exposure risk in China. Three vaccination doses were freely provided to all residents: December 2020 for the first dose, March 2021 for the second, and December 2021 for the third. The inactivated vaccine booster dose is recommended 3 months after the first dose and 6 months after the second dose. Data from vaccination records were confirmed online using a national government service platform. We gathered adverse events of SARS-CoV-2 vaccines among study participants using biologic agents to confirm safety-related issues (Supplementary Table 3).

As no statistical distinction in the protection efficacy among different types of inactivated SARS-CoV-2 vaccines was reported (21), we distinguished vaccination status based on dose. According to the World Health Organization Strategic Advisory Group of Experts on Immunization (SAGE), inactivated SARS-CoV-2 vaccines should be injected at least twice to maintain efficacy. Three doses were indicated for vaccination, with all available booster doses. Finally, there were 175 patients who were unvaccinated, 31 patients received one dose, 166 patients received two doses, and 164 patients received three vaccination doses.

### 2.3 Outcomes

The primary outcome was the presence of any new-onset GI symptoms after SARS-CoV-2 infection before a negative SARS-CoV-2 nucleic acid test or a negative self-test for antigens. Previous literature on infected patients showed that approximately 15% of them had GI symptoms after COVID-19 infections, indicating that GI symptoms were common in patients with COVID-19 (10). Therefore, new-onset GI symptoms could be attributed to acute Omicron infections in patients with IBD. GI symptoms, including anorexia, abdominal pain, diarrhea, nausea or vomiting, hematochezia, and increased bowel movements, were recorded in the questionnaire (Supplementary material 1). We combined nausea and vomiting, as they are upper GI symptoms and often occur together. Previous reports indicated anorexia was the most frequent GI symptom after COVID-19 (22, 23). We defined anorexia with other GI symptoms as “anorexia as comorbidity” because a single anorexia symptom is not a strong indicator of a GI-symptomatic infection. The secondary outcomes were anorexia as a comorbidity, abdominal pain, diarrhea, nausea or vomiting, hematochezia, and increased bowel movements. Supplementary Table 4 lists detailed information on the participants with a single symptom of anorexia after infection.

### 2.4 Covariates

We incorporated multiple covariates, including age, sex, body mass index (BMI, continuous), the adapted Charlson Comorbidity Index (CCI), smoking status (never, past, or current), IBD duration (continuous), IBD type (CD or UC), IBD status (remission or active), currently the use of 5-aminosalicylic acids (5-ASA), corticosteroid, immunosuppressors, anti-tumor necrosis factor agents (anti-TNF), anti-α4β7-integrins agents, anti-interleukin-12/interleukin-23, and COVID severity (asymptomatic, mild illness, moderate illness, and severe illness).

The adapted CCI considered age and various comorbidities, including cardiovascular and cerebrovascular disease, chronic lung diseases, chronic liver disease, kidney disease, diabetes, and tumors (Supplementary Table 5). Categorized as 0–1, 2–3, or 4+, it served as an indicator of the overall comorbidity burden (24). Supplementary Table 6 lists the detailed comorbidity information for the study population. IBD status was defined as the disease activity in the week preceding the COVID-19 infection, assessed using questionnaire forms (Supplementary Section 4). For patients with CD, disease activity was determined based on the best Crohn's Disease Activity Index (CDAI) score (25), where total scores < 150 indicate remission. For UC patients, disease status is evaluated using the severity score according to the Truelove and Witts criteria (26). Regarding COVID-19 severity, we referred to the “Diagnosis and treatment protocol for COVID-19 patients (Tentative 10th Version in China)” (27). Mild illness pertained to patients with positive nucleic acid or antigen tests for COVID-19 and an upper respiratory tract infection. Patients with moderate illness had prolonged fever (>3 days) and radiological evidence of pneumonia. A severe illness was characterized by hospitalization.

### 2.5 Primary statistical analysis

Descriptive statistics for exposure variables were presented as mean and standard deviation (SD) for continuous data and as counts and percentages for categorical data. Data were stratified based on vaccination doses, and statistical comparisons were performed using the Kruskal-Wallis test for continuous data and the χ^2^ or Fisher exact test for categorical data (Table 2).

**Table 2:** The characteristics of the study population, stratified by vaccination doses.

**Factor**	**Unvaccinated (*N =* 175)**	**One dose (*N =* 31)**	**Two doses (*N =* 166)**	**Three doses (*N =* 164)**	***P*-value**
**Sex**					
Male	91 (52.0%)	23 (74.2%)	98 (59.0%)	115 (70.1%)	0.003
Female	84 (48.0%)	8 (25.8%)	68 (41.0%)	49 (29.9%)	
Age (years)	40.4 (14.0)	39.9 (15.0)	35.5 (12.8)	39.8 (14.0)	0.004
BMI (kg/m^2^)	21.7 (3.0)	20.7 (3.0)	21.8 (3.7)	22.1 (3.8)	0.193
**Smoking status**					
Never	146 (83.4%)	25 (80.6%)	137 (82.5%)	128 (78.0%)	0.835
Past	25 (14.3%)	5 (16.1%)	22 (13.3%)	29 (17.7%)	
Current	4 (2.3%)	1 (3.2%)	7 (4.2%)	7 (4.3%)	
**Adapted CCI**					
0–1	146 (83.4%)	23 (74.2%)	153 (92.2%)	138 (84.1%)	0.064
2–3	17 (9.7%)	6 (19.4%)	10 (6.0%)	17 (10.4%)	
4+	12 (6.9%)	2 (6.5%)	3 (1.8%)	9 (5.5%)	
**COVID severity**					
Asymptomatic	8 (4.6%)	0 (0%)	10 (6.0%)	14 (8.5%)	0.538
Mild illness	155 (88.6%)	30 (96.8%)	149 (89.8%)	145 (88.4%)	
Moderate illness	9 (5.1%)	1 (3.2%)	6 (3.6%)	4 (2.4%)	
Severe illness	3 (1.7%)	0 (0%)	1 (0.6%)	1 (0.6%)	
IBD duration (years)					0.009
< 5	59 (33.7%)	8 (25.8%)	65 (39.2%)	78 (47.6%)	
5–10	63 (36.0%)	17 (54.8%)	68 (41.0%)	60 (36.6%)	
>10	53 (30.3%)	6 (19.4%)	33 (19.9%)	26 (15.9%)	
**IBD type**					
CD	146 (80.7%)	26 (81.3%)	127 (73.4%)	115 (67.3%)	0.065
UC	35 (19.3%)	6 (18.8%)	46 (26.6%)	56 (32.7%)	
**IBD status**					
Remission	137 (78.3%)	22 (71.0%)	121 (72.9%)	133 (81.1%)	0.466
Active	38 (21.7%)	9 (29.0%)	45 (27.1%)	31 (18.9%)	
**IBD medication group**					
5-ASA	27 (15.4%)	3 (9.7%)	30 (18.1%)	48 (29.3%)	0.004
Corticosteroid	5 (2.9%)	1 (3.2%)	3 (1.8%)	5 (3.0%)	0.916
MTX/AZA	25 (14.3%)	3 (9.7%)	11 (6.6%)	14 (8.5%)	0.104
Anti-TNF	47 (26.9%)	9 (29.0%)	49 (29.5%)	33 (20.1%)	0.237
Anti-α4β7-integrins	13 (7.4%)	2 (6.5%)	9 (5.4%)	8 (4.9%)	0.787
Anti-IL-12/IL-23	18 (10.3%)	6 (19.4%)	21 (12.7%)	14 (8.5%)	0.282

Variables were described using mean (SD) and n (%), as appropriate. BMI, body mass index; IBD, inflammatory bowel disease; CD, Crohn's disease; UC, ulcerative colitis; CCI, Charlson comorbidity index; AZA, azathioprine; MTX, methotrexate; TNF, tumor necrosis factor; IL, interleukin.

The association between vaccination dose and new-onset GI symptoms after infection was estimated by comparing the odds ratios (ORs) of three, two, or one vaccination doses compared with the unvaccinated group using unadjusted and adjusted logistic regressions. The unadjusted model contained only the exposure factor (vaccination dose) and primary outcomes (new-onset GI symptoms following infection). The adjusted model also contained confounders, including age, sex, BMI, adapted CCI, smoking status, IBD duration, IBD type, IBD status, and IBD medications (13, 28). ORs were estimated in each vaccination dose group compared with the unvaccinated group, with lower ORs suggesting more protective efficacy.

Sensitivity analyses were performed using the matched groups of propensity scores. The matched groups were well balanced for critical baseline variables (COVID-19 severity and IBD type; Supplementary Table 7). The association between the vaccination dose (0 or three doses) and GI symptoms was re-estimated using logistic regression models.

Two-sided 95% confidence intervals (CIs) were calculated for each reported OR, with 95% CIs that excluded 1 considered statistically significant. Two-sided *p*-values were tested and reported. The *p*-values for trends were estimated to evaluate the dose-response effect of vaccine doses (29).

### 2.6 Subgroup analysis

We conducted subgroup analyses defined by sex, age, IBD duration (< 5 years, 5–10 years, or >10 years), BMI (< 18.5 kg/m^2^, 18.5–24 kg/m^2^, or >24 kg/m^2^), IBD type, IBD status, smoking status, adapted CCI group, COVID severity, and IBD medications comparing between the group of three vaccination doses and the unvaccinated group. Multivariable logistic regression models were used, adjusting for all the confounders as mentioned before. We also used Firth's penalized logistic regression for subgroups with case numbers < 60 to minimize bias in effect sizes (30). Subgroup analyses stratified based on sex, age, BMI, smoking status, adapted CCI, COVID severity, IBD duration, IBD type, and IBD status were also performed in matched 270 participants with IBD. We further conducted a multiplicative interaction analysis in the subgroup analysis to determine the interaction between the three vaccination doses and each confounder. These final subgroup multivariate models reported adjusted odds ratios (aORs) and 95% CIs.

### 2.7 Secondary analysis

For secondary outcomes of each GI symptom following infection, descriptive outcome data were compared using the χ^2^ or Fisher exact test (**Table 4**). Multivariate logistic regression analyses were used to identify the vaccination doses associated with each GI symptom. Other GI symptoms were excluded from our secondary analysis because of variant behaviors and the small number of cases (*N* = 10). The models for these outcomes were adjusted for the same confounders as those used in the adjusted logistic model. The frequencies of all new-onset GI symptoms in the different vaccination dose groups were plotted. All statistical analysis in this study were performed using the R software (version 4.2.1).

## 3 Results

### 3.1 Participant characteristics

In total, 702 participants completed the questionnaires. Notably, 536 (61% were male, mean age was 38.6 years [SD 13.79], and 74.4% had Crohn's disease) reported SARS-CoV-2 infection for the first time between October 2022 and February 2023 and were included in the study. Among them, 175 (32.6%) were unvaccinated, 31 (5.8%) received one dose of the inactivated SARS-CoV-2 vaccine, 166 (31.0%) received two doses, and 164 (30.6%) received three doses of the inactivated SARS-CoV-2 vaccine at least 1 month before the infection (Table 2).

Patients with three vaccination doses were significantly more male subjects (70.1% vs. 52.0% of the unvaccinated group, *P* < 0.01), were slightly younger (mean [SD]: 39.8 [14.0] vs. 40.4 [14.0] of the unvaccinated group), were more patients with UC (32.7% vs. 19.3% of the unvaccinated group, *P* < 0.05), had shorter IBD duration time (*P* < 0.01), and used more 5-ASA (29.2% vs. 15.5% of the unvaccinated group, *P* < 0.01).

The four vaccination status groups showed no significant differences in the adapted CCI, BMI, smoking status, IBD status, COVID-19 severity, or other medications (except 5-ASA). Table 2 and Supplementary Table 4 present the full details regarding demographics, IBD clinical characteristics, COVID-19 severity, IBD medications, and adapted CCI.

### 3.2 Association between vaccination doses and GI symptoms after SARS-CoV-2 infection

In the study group of 536 patients (76.4% of all 702 participants), 262 (48.9% of the 536 patients with COVID-19 infection) reported acute GI symptoms (anorexia, abdominal pain, diarrhea, nausea, vomiting, hematochezia, and increased bowel movements) following SARS-CoV-2 infection. Notably, 94 (53.7%) unvaccinated patients had GI symptoms. For patients who received one, two, or three doses of vaccination, the number of individuals with GI symptoms was 17 (54.8% of 31 participants), 88 (53.0% of 166 participants), and 62 (37.8% of 164 participants), respectively.

Table 3 shows the association between vaccination dose and GI symptoms in 536 patients. In the unadjusted logistic model, the group of three vaccination doses was associated with a 48% lower occurrence rate (OR = 0.52, 95% CI 0.32–0.81, *P* = 0.0035) of GI symptoms compared with the unvaccinated group. Multivariate logistic regression analysis also confirmed that three vaccination doses were associated with fewer GI symptoms than the unvaccinated (aOR = 0.56, 95% CI 0.34–0.90, *P* = 0.018). The aORs for GI symptoms in patients who received one or two doses of vaccination were 0.86 (95% CI 0.37–2.00, *P* = 0.73) and 0.97 (95% CI 0.61–1.55, *P* = 0.90), respectively, compared with the unvaccinated group. A decreasing trend in the aORs for reduced GI symptoms with increasing vaccination doses was confirmed (*P-*value for trend = 0.039).

**Table 3:** Association of vaccination doses and the occurrence of GI symptoms in 536 patients.

**Group**	**Vaccination status**	**GI symptomatic positive/negative cases**	**OR (95% CI)**		***P*-value ^a^**	***P* for trend ^b^**
			**Unadjusted**	**Adjusted** ^c^		
IBD (*N =* 536)	Unvaccinated	94/81	Ref	Ref	Ref	0.039
	One dose	17/14	1.05 (0.49–2.28)	0.86 (0.37–2.00)	0.73	
	Two doses	88/78	0.97 (0.63–1.49)	0.97 (0.61–1.55)	0.90	
	Three doses	62/102	0.52 (0.34–0.81)^*^	0.56 (0.34–0.90)	0.018	
CD (*N =* 399)	Unvaccinated	76/64	Ref	Ref	Ref	0.68
	One dose	13/13	0.84 (0.36–1.96)	0.75 (0.30–1.86)	0.54	
	Two doses	67/56	1.01 (0.62–1.64)	0.97 (0.58–1.64)	0.92	
	Three doses	43/67	0.54 (0.32–0.89)^**^	0.54 (0.31–0.94)	0.029	
UC (*N =* 137)	Unvaccinated	18/17	Ref	Ref	Ref	0.40
	One dose	4/1	3.78 (0.50–77.93)	3.67 (0.30–96.34)	0.34	
	Two doses	21/22	0.90 (0.37–2.20)	0.69 (0.21–2.17)	0.53	
	Three doses	19/35	0.51 (0.21–1.22)	0.67 (0.22–2.03)	0.48	

OR, odds ratio, GI symptom, gastrointestinal symptom; IBD, inflammatory bowel disease; CD, Crohn's disease; UC, ulcerative colitis. ^a^*P*-value for the comparison of the adjusted OR for one dose vs. unvaccinated, two doses vs. unvaccinated, or three doses vs. unvaccinated. ^b^P for trend for the dose-response effect of the adjusted ORs for 1, 2, and 3 doses vs. unvaccinated. ^c^An adjusted OR < 1 indicated that receipt of 1, 2, or 3 doses (vs. unvaccinated) was less likely among GI symptom-positive cases vs. GI symptom-negative cases. Models included age, sex, BMI, adapted CCI (0–1, 2–3, and 4+), smoking status, IBD duration, IBD type, IBD status, and IBD medications (Table 2). ^*^ *P* = 0.0035. ^**^ *P* = 0.017.

A sensitivity analysis was performed to confirm the correlation between vaccination doses and reduced GI symptoms. The association between vaccination doses and reduced GI symptoms was consistent in the matched 270 patients with IBD (three doses vs. the unvaccinated group, OR= 0.53, 95% CI 0.30–0.93, *P* = 0.027) (Supplementary Table 8).

### 3.3 Subgroup analysis

**Figure 2:**
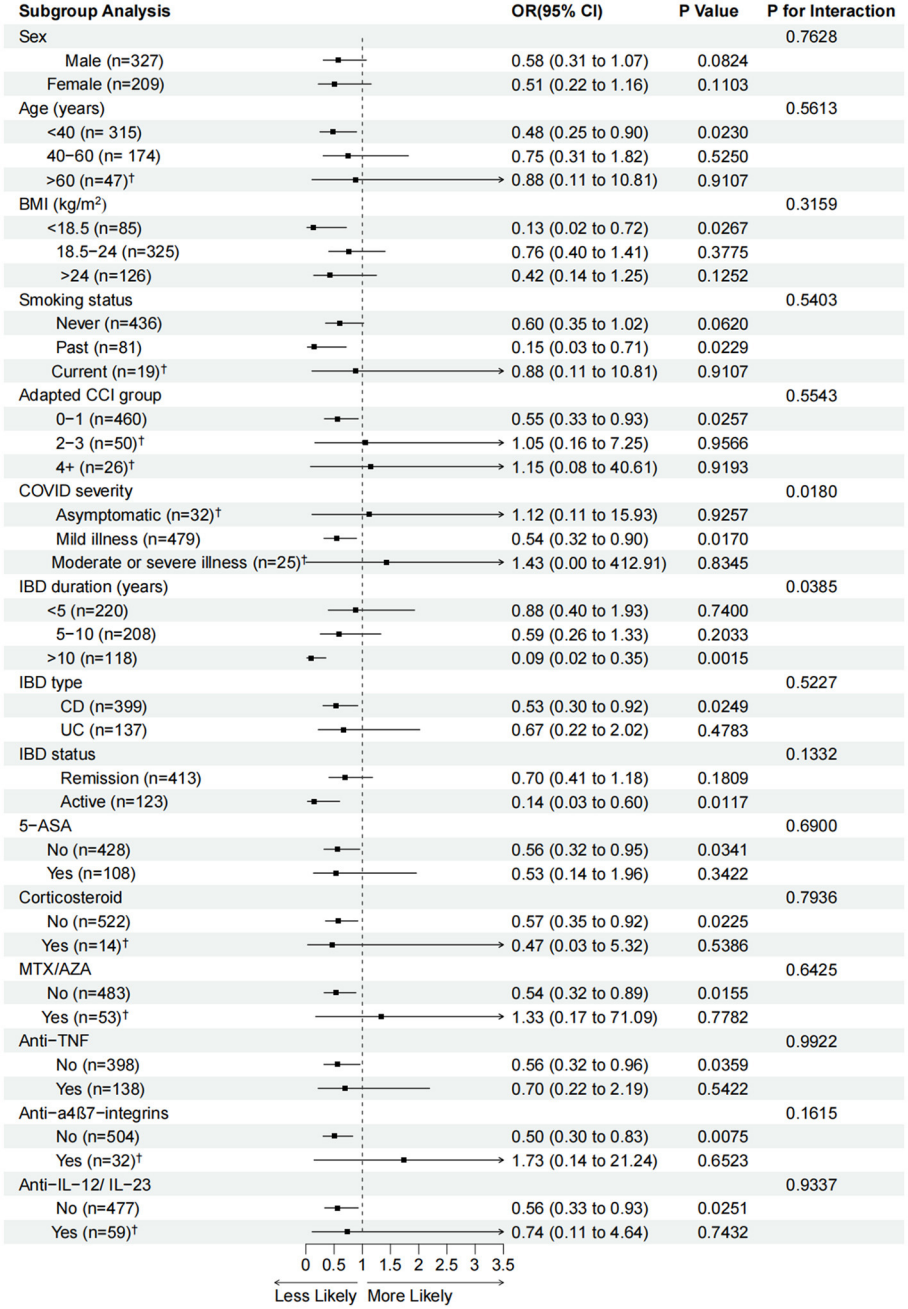
Adjusted odds ratios (aORs, 95% confidence intervals) for GI-symptomatic infections associated with three vaccination doses in each subgroup compared with the unvaccinated group. aORs were calculated using logistic regression models after adjusting for age, sex, BMI, adapted CCI, smoking status, IBD duration, IBD type, IBD status, and IBD medications. OR (95% CI), *P*-values and P for interaction are calculated in the adjusted model. BMI, body mass index; IBD, inflammatory bowel disease; CD, Crohn's disease; UC, ulcerative colitis; CCI, Charlson comorbidity index; AZA, azathioprine; MTX, methotrexate; TNF, tumor necrosis factor; IL, interleukin. ^†^Logistic regressions with Firth-type penalization were used to analyze these subgroups with <60 cases.

Figure 2 shows the subgroup analysis results between the three vaccination doses and GI symptomatic infections compared with the unvaccinated group. Regarding demographic characteristics, the protective efficacy of the three doses of vaccination was significant in patients aged < 40 years (OR 0.48, 95% CI 0.25–0.90), BMI < 18.5 kg/m^2^ (OR 0.13, 95% CI 0.02–0.72), with past smoking status (OR 0.15, 95% CI 0.03–0.71), and adapted CCI 0–1 (OR 0.55, 95% CI 0.33–0.93) compared with the unvaccinated group. As for COVID-19 severity, the three vaccination doses group was associated with reduced GI symptoms in those with mild illness (OR 0.54, 95% CI 0.32–0.90) compared with the unvaccinated group. In IBD clinical characteristics, we found robust protective efficacy in patients with IBD duration >10 years (OR 0.09, 95% CI 0.02–0.35), CD (OR 0.53, 95% CI 0.30–0.92), and active IBD status (OR 0.14, 95% CI 0.03–0.60) compared with the unvaccinated group. The protective efficacy was consistent within each subgroup according to IBD duration, type, and status. For IBD medications, the subgroups with no drug use were more likely to be significantly associated with the protective efficacy of the three vaccination doses vs. the unvaccinated group. In the matched 270 participants, the subgroup analysis results were consistent with those of the unmatched participants with the adapted CCI 0–1 (OR 0.50, 95% CI 0.28–0.89), COVID-19 severity of mild illness (OR 0.53, 95% CI 0.30–0.92), IBD duration >10 years (OR 0.13, 95% CI 0.07–0.89), CD (OR 0.48, 95% CI 0.26–0.90), and active IBD status (OR 0.14, 95% CI 0.02–0.67) (Supplementary Figure 2).

The interactions between the three vaccination doses group and all confounders were tested. The results showed a significant interaction in the subgroups of IBD duration (*P* = 0.0385) and COVID-19 severity (*P* = 0.0180).

### 3.4 Secondary outcome analysis

**Figure 3:**
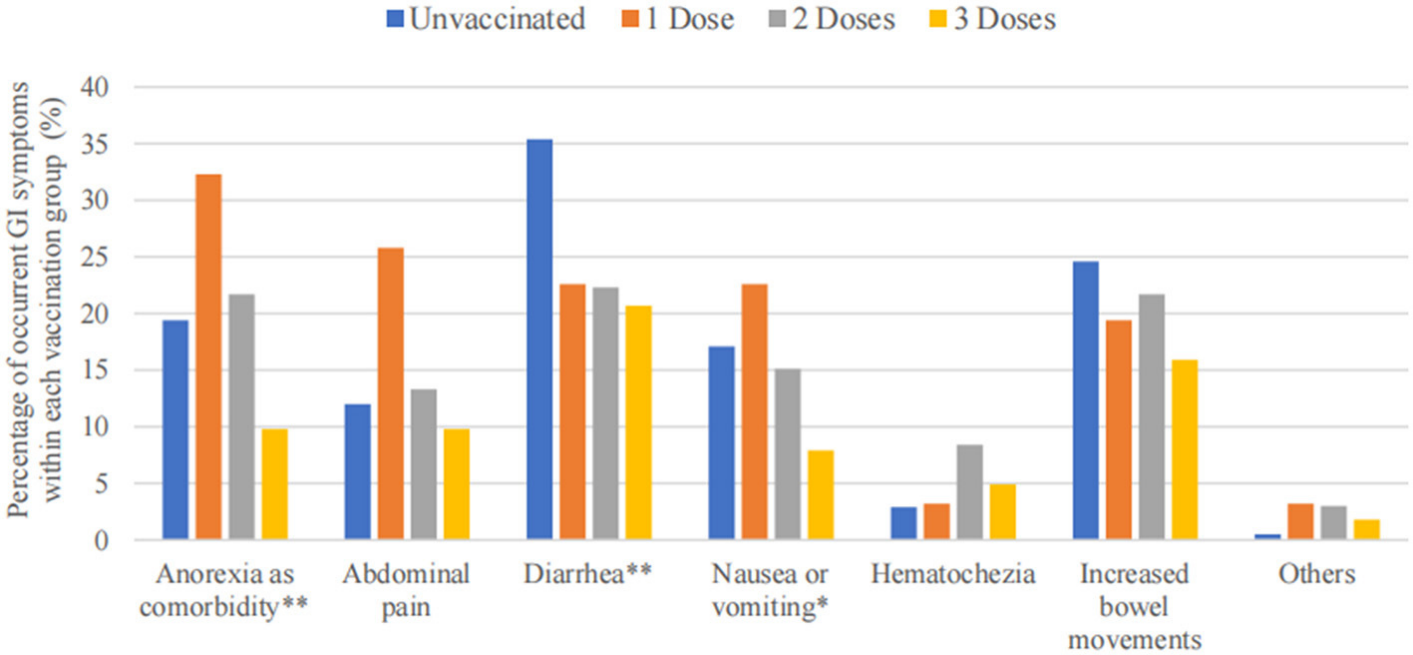
Bar graph of GI symptoms among 536 patients, stratified based on vaccination doses. ** *P* < 0.01, * *P* < 0.05.

The three most frequent GI symptoms following COVID-19 infection in this study were diarrhea (140 in 536 participants, 26.1%), increased bowel movements (111 in 536 participants, 20.7%), and anorexia (96 in 536 participants, 17.9%). Patients who received the three vaccination doses reported lower occurrences of various GI symptoms, especially anorexia (*P* < 0.01), diarrhea (*P* < 0.01), and nausea or vomiting (*P* < 0.05) (Table 4). Figure 3 shows the decreasing trend in symptomatic GI infections among the four vaccination dose groups for each GI symptom.

**Table 4:** The occurrence of various GI symptoms in 536 patients, stratified by vaccination doses.

**Types of GI symptoms**	**Unvaccinated (*N =* 175)**	**One dose (*N =* 31)**	**Two doses (*N =* 166)**	**Three doses (*N =* 164)**	***P*-value**
Anorexia as comorbidity	34 (19.4%)	10 (32.3%)	36 (21.7%)	16 (9.8%)	0.003
Abdominal pain	21 (12.0%)	8 (25.8%)	22 (13.3%)	16 (9.8%)	0.097
Diarrhea	62 (35.4%)	7 (22.6%)	37 (22.3%)	34 (20.7%)	0.008
Nausea or vomiting	30 (17.1%)	7 (22.6%)	25 (15.1%)	13 (7.9%)	0.036
Hematochezia	5 (2.9%)	1 (3.2%)	14 (8.4%)	8 (4.9%)	0.126
Increased bowel movements	43 (24.6%)	6 (19.4%)	36 (21.7%)	26 (15.9%)	0.253
Other GI symptoms	1 (0.6%)	1 (3.2%)	5 (3.0%)	3 (1.8%)	0.380

Variables were described using *n* (%).

**Figure 4:**
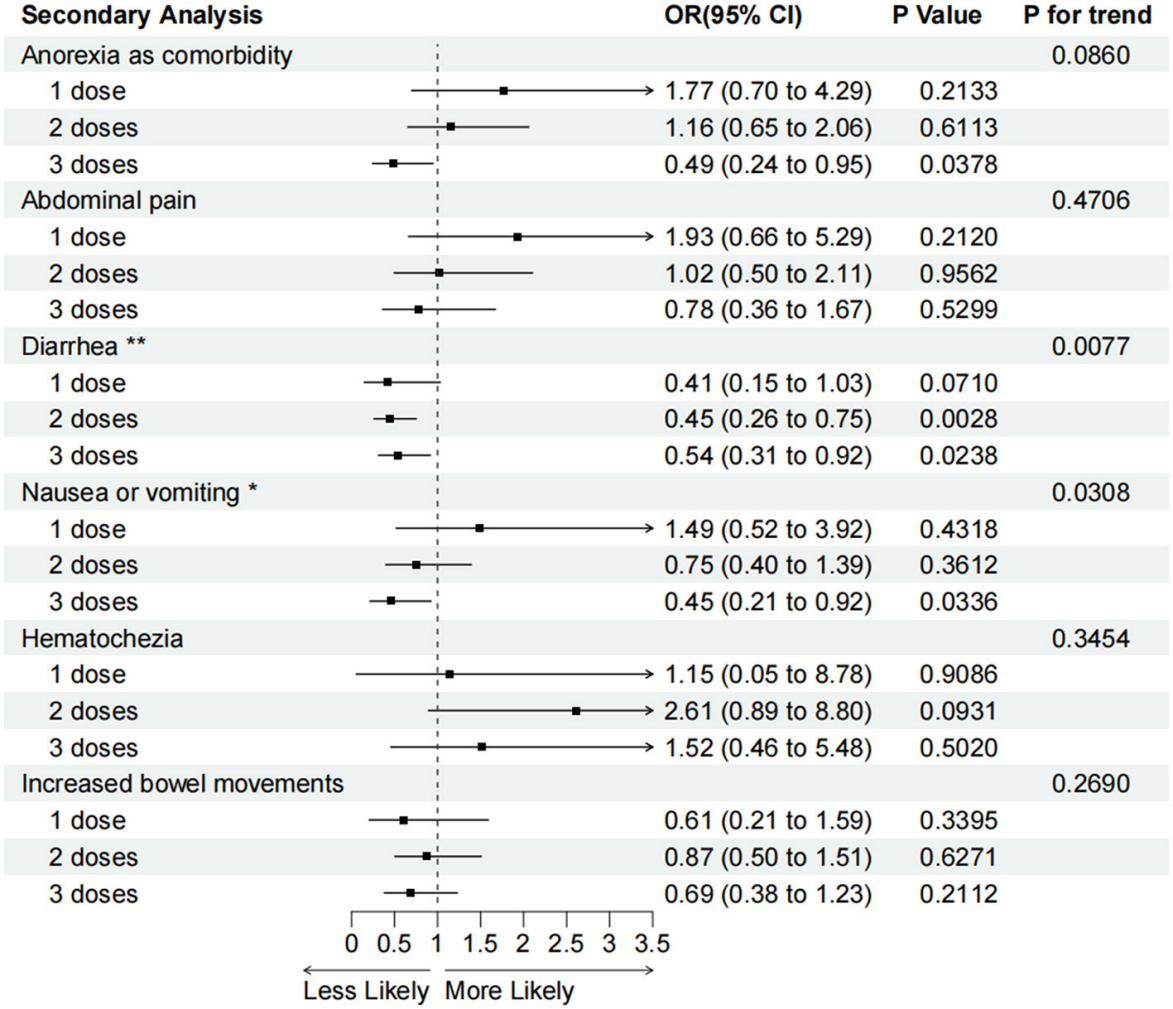
Adjusted odds ratios (95% confidence intervals) for various GI symptoms associated with one, two, or three vaccination doses compared with the unvaccinated. OR (95% CI), *P*-values and P for interaction are calculated in the adjusted model. *P for trend < 0.05, **P for trend <0.01.

After adjusting for all the confounders, we found that in the 536 patients with IBD, two or three vaccination doses reduced diarrhea compared with the unvaccinated group (two doses group: aOR = 0.45, 95% CI 0.26–0.75, *P* < 0.01; three doses group: aOR = 0.54, 95% CI 0.31–0.92, *P* < 0.05). The protective efficacy of vaccination for anorexia and nausea or vomiting in patients who received three vaccination doses was also confirmed (anorexia: aOR = 0.49, 95% CI 0.24–0.95, *P* < 0.05; nausea or vomiting: aOR = 0.45, 95% CI 0.21–0.92, *P* < 0.05). However, the association between one, two, or three vaccination doses and reduced abdominal pain, hematochezia, and increased bowel movements was not statistically significant (Figure 4).

A secondary analysis of multivariable logistic regression also showed that higher doses of vaccination were correlated with a lower occurrence of diarrhea (*P* for trend < 0.01) and nausea or vomiting (*P* for trend < 0.05) compared with the unvaccinated group (Figure 4).

## 4 Discussion

Our study has demonstrated an association between the three vaccination doses and reduced GI symptoms during COVID-19 infection in a population representative of patients with IBD from three tertiary hospitals in eastern China. Compared with the unvaccinated group, the three vaccination doses for patients with active IBD status and >10 years of disease duration showed a protective effect against symptomatic GI infection.

A dose-response effect was observed between an increased vaccination dose and decreased GI symptoms. However, the protective effects of one or two vaccination doses were not statistically significant. These findings were consistent with recent data from Hong Kong (12), which showed that three doses of inactivated vaccines (CoronaVac) provided a high level of protection against severe infection-caused disease. However, two-dose inactivated vaccine recipients showed reduced vaccine effectiveness. Vaccination protection against symptomatic GI infections may depend on a complete vaccination with all booster doses. We also found that higher vaccination doses were correlated with less diarrhea, nausea, and vomiting.

Previous studies have raised concerns about vaccine efficacy in patients using immunosuppressant medications. Available data for the World Health Organization Emergency Use Listing COVID-19 vaccine products suggest that vaccine effectiveness and immunogenicity are lower in patients using immunosuppressant medications than in other patients. Cheung et al. demonstrated that the third and fourth COVID-19 vaccine doses broadened and prolonged immunity against SARS-CoV-2 in adult patients with immune-mediated inflammatory diseases (31, 32). Consistent with previous findings, we found that the protective efficacy of the three vaccination doses was relatively insignificant in patients using immunosuppressors. Individuals receiving immunosuppressive therapy may require additional vaccine doses for protection.

Several factors may have contributed to the association between vaccination and reduced GI symptoms. First, COVID-19 enters the GI mucosa by binding to angiotensin-converting enzyme 2 (ACE2) receptors, which are widely expressed in the GI tract (33, 34). COVID-19 disrupts the normal physiological function of ACE2, including regulating glucose transporter proteins, leading to symptoms such as diarrhea, nausea, vomiting, and abdominal pain (35). Vaccination against COVID-19 stimulates the immune system to produce antibodies that can neutralize the virus, thereby reducing its ability to invade and replicate within the gastrointestinal tract, consequently mitigating the severity of associated symptoms (36). Second, the COVID-19 infection might be associated with an exacerbation of anxious-depressive symptoms (8) and potentially a reduction in therapeutic adherence in IBD patients (9). Emotional fluctuations during the pandemic could be reduced by COVID vaccination, potentially reducing GI symptoms via alleviating disease activity in patients with IBD. Another potential mechanism of the GI symptoms triggered by COVID-19 infection is the gut microbiota alteration, which may contribute to immune dysfunction and severe disease in COVID-19-infected patients (37). The gut microbiota alteration exists in the intestine of patients with COVID-19 compared with healthy individuals and is characterized by an increased abundance of bacteremia-associated bacteria and a decreased level of symbionts, which contribute to host immunity. This disturbance persists for a long time, even if respiratory manifestations disappear and the throat swab turns negative (38, 39). It remains unknown whether the effect of vaccination against GI symptoms is associated with the protection of the gut microbiota.

This study possesses several notable strengths. The first is the study's focus on the efficacy of vaccination against GI symptoms in patients with IBD. Patients with IBD face an elevated risk of experiencing new-onset GI symptoms triggered by COVID-19, which may exacerbate IBD flares or worsen disease progression (4, 5, 10). However, there has been limited attention from researchers to the epidemic evidence of new-onset GI symptoms following the COVID-19 infection. Our research addresses this gap and confirms the protective efficacy of the SARS-CoV-2 vaccination among patients with IBD. Second, we employed propensity score matching analysis to mitigate potential biases inherent in the retrospective nature of the study. The association between vaccination doses (0 or 3 doses) and the occurrence of GI symptoms was found to be significant in the matched cohort of 270 IBD patients (3 doses vs. unvaccinated: OR = 0.53, 95% CI 0.30–0.93, *P* = 0.027). Third, recognizing Crohn's disease and ulcerative colitis as different types of IBD, we conducted separate analyses based on IBD subtypes in both matched and unmatched patient groups. Our results indicated that among patients with Crohn's disease, receiving three doses of the vaccine was significantly associated with fewer GI symptoms following COVID-19 infection compared to those who did not receive the vaccine. Furthermore, in subgroup analyses, we identified interaction modifiers (COVID severity and IBD duration) between vaccination doses and reduced GI symptoms. It is plausible that vaccination efficacy was more pronounced in patients with mild COVID-19 illness and a longer duration of IBD, though further studies are warranted to elucidate and confirm this observation. Additionally, our findings revealed that receiving all three doses of the vaccine was associated with a reduced incidence of new-onset GI symptoms among patients with active IBD status, with consistent results observed in sensitivity analyses, indicating a significant role of vaccination in reducing GI symptoms following COVID-19 infection among patients with active disease status.

This study has some limitations. First, due to regional constraints, our data collection was limited to patients administered with inactivated vaccines. Although we observed a correlation between post-COVID-infected IBD patients who received three doses of the inactivated protein vaccine and reduced GI symptoms, generalizing this association to IBD patients vaccinated with other types of vaccines in different regions poses challenges. Different types of vaccines, including mRNA-, viral-, inactivated-, and protein-based vaccines, have been widely used in various regions. Large-scale clinical studies on the protective efficacy of different vaccines against COVID-19, particularly in alleviating GI symptoms in patients with IBD, are still lacking. Inactivated protein vaccines demonstrate excellent safety, with minimal adverse events reported among IBD patients receiving them (40). In our study, IBD patients receiving inactivated vaccines showed minimal occurrence of adverse events, suggesting that administering three vaccine doses to these patients could offer greater benefits. Second, the small sample size in the one-dose vaccination group (31 cases) compared to the unvaccinated group (175 cases) could introduce bias into our results. It may affect the statistical power to identify associations within this subgroup. Hence, caution is advised in interpreting findings related to the one-dose vaccination group. Nevertheless, receiving two or three doses or choosing not to be vaccinated is the prevailing pattern in society, with few people opting for a single-dose vaccination. Notably, patients who received fewer vaccine doses expressed concerns about vaccination exacerbating their condition in the survey. However, our study proved that receiving more vaccine doses did not exacerbate IBD symptoms but offered further protection. This adds significant clinical relevance to our research. Third, potential selection bias may have influenced our results, as recruited IBD patients may differ in treatment regimens, characteristics, or experiences compared to patients in other regions. Finally, recall bias is another concern. To address these limitations, we implemented rigorous data collection methods, including validating self-reported vaccination status and COVID-19 onset dates where feasible. We recorded COVID-19 onset dates for all participants, indicating a recent infection and minimizing recall bias. Nonetheless, we recommend future studies to adopt diverse data collection methods to mitigate self-reporting biases further.

## 5 Conclusion

We found that a three-dose vaccination status, but not one or two doses, was associated with reduced GI symptoms after SARS-CoV-2 infection compared with an unvaccinated status. A dose-response effect was observed across the one, two, and three vaccination doses. The three vaccination doses showed significant protection in patients with CD, duration of >10 years, and active disease status. In addition, the association between two or three vaccination doses and the reduced occurrence of diarrhea was significant. We also confirmed that the three vaccination doses were correlated with fewer occurrences of diarrhea, nausea, and vomiting following infection.

Given the efficacy demonstrated by complete vaccination status, patients with IBD, especially those with longer disease duration and active disease status, may benefit from considering booster doses as part of their ongoing healthcare strategy.

## Data availability statement

The raw data supporting the conclusions of this article will be made available by the authors, without undue reservation.

## Ethics statement

The studies involving humans were approved by the Ethics Committee of Ruijin Hospital, Shanghai Jiao Tong University School of Medicine. The studies were conducted in accordance with the local legislation and institutional requirements. The participants provided their written informed consent to participate in this study.

## Author contributions

YH: Conceptualization, Data curation, Formal analysis, Methodology, Writing – original draft, Writing – review & editing. TC: Data curation, Formal analysis, Methodology, Writing – original draft, Writing – review & editing, Investigation. XS: Data curation, Writing – review & editing. JC: Conceptualization, Writing – review & editing. KW: Investigation, Writing – review & editing. LZ: Investigation, Writing – review & editing. WGa: Investigation, Writing – review & editing. YZ: Writing – review & editing. WGe: Data curation, Formal analysis, Methodology, Writing – review & editing. YG: Conceptualization, Writing – review & editing. DZ: Writing – review & editing.

## Supplementary material

Supplementary Figure 1 Propensity score distribution in the non-exposed and exposed groups. The non-exposed stands for the unvaccinated, the exposed stands for the 3 vaccination doses group.

Supplementary Figure 2 Adjusted odds ratios (aORs, 95% confidence intervals) for GI symptomatic infections associated with three vaccination doses in each subgroup vs. the unvaccinated in the matched 270 participants. aORs were calculated using logistic regression models after adjustments for age, sex, BMI, adapted CCI, smoking status, IBD duration, IBD type and IBD status. OR (95% CI), *P*-values, and P for interaction are calculated in the adjusted model. BMI, body mass index; IBD, inflammatory bowel disease; CD, Crohn's disease; UC, ulcerative colitis; CCI, Charlson comorbidity index.^†^ Logistic regressions with Firth-type penalization were used to analyze these subgroups with case number < 60.

Supplementary Table 1 Characteristics of 116 patients with negative or indetermined infection status.

Supplementary Table 2 Characteristics of 21 patients excluded in the study with confirmed infection status.

Supplementary Table 3 Self-reported adverse events of 152 vaccinated IBD patients using biology agents in the study population.

Supplementary Table 4 Detailed information of the study population with single anorexia after infection.

Supplementary Table 5 Score table of adapted Charlson comorbidity index in the study.

Supplementary Table 6 Detailed comorbidity information of the study population.

Supplementary Table 7 Baseline characteristics of participants of unvaccinated and 3 doses vaccination after propensity score matching.

Supplementary Table 8 Association of vaccination doses and the occurrence of GI symptoms among 270 patients after propensity score matching.
