# Supplementary material for: The association of three vaccination doses with reduced gastrointestinal symptoms after severe acute respiratory syndrome coronavirus 2 infections in patients with inflammatory bowel disease
Source: Front Med (Lausanne). 2024 Mar 18;11:1377926. doi: 10.3389/fmed.2024.1377926 (PMC10982480; doi:10.3389/fmed.2024.1377926)
Supplement: Supplementary file 1 [file Data_Sheet_1.docx]

Survey on the Incidence of COVID-19 Infection in Patients with Inflammatory Bowel Disease

Dear patient, thank you for participating in this survey. The study protocol was approved by the ethics committee of Ruijin Hospital, Shanghai Jiao Tong University School of Medicine (ID: 2023-167). Submitting this questionnaire is considered as signing the informed consent form. The data from this project will be used exclusively for research purposes and will not involve any privacy exposure issues. Please feel free to complete the questionnaire.

Section 1: Basic Information

1. Your Gender [Single-choice Question] *

○ Male ○ Female

2. Year of Birth (Enter the year, e.g., 1978) [Open-ended Question] *

3. What is your height (in centimeters, round to the nearest whole number, e.g., 175)? [Open-ended Question] *

4. What is your weight (in kilograms, round to the nearest whole number, e.g., 65)? [Open-ended Question] *

5. In which year were you diagnosed with inflammatory bowel disease (Enter the year, e.g., 2018)? [Open-ended Question] *

6. What is the type of inflammatory bowel disease your are diagnosed? [Single-choice Question] *

○ Crohn's Disease

○ Ulcerative Colitis

7. If diagnosed with Crohn's Disease, where is the location of your lesions? [one or more answer Question] *

□ Ileum

□ Colon

□ Ileum and Colon

□ Upper digestive tract (Esophagus, Stomach, Duodenum, Jejunum)

□ Not sure

(Note: Question 7 is dependent on the first option selected in Question 6.)

8. If diagnosed with Ulcerative Colitis, what is the extent of involvement in your case? [Single-choice Question] *

○ Proctitis

○ Left-sided Colitis

○ Pancolitis

○ Not sure

(Note: Question 8 is dependent on the second option selected in Question 6.)

9. Are you currently having active symptoms of inflammatory bowel disease? (Active phase refers to the presence of symptoms such as abdominal pain, diarrhea, urgency, abdominal mass, systemic symptoms, etc.) [Single-choice Question] *

○ Yes

○ No

○ Not sure

10. Have you ever been diagnosed with or are currently diagnosed with the following diseases? [One or more choice Question] *

□ Cardiovascular diseases (such as hypertension, myocardial infarction, etc.)

□ Cerebrovascular diseases (such as stroke, cerebral hemorrhage, etc.)

□ Chronic respiratory diseases (such as chronic bronchitis, bronchiectasis, etc.)

□ Chronic liver diseases

□ Kidney diseases (such as chronic kidney disease, uremia)

□ Diabetes

□ Tumors

□ Other _________________*

□ None

11. Do you smoke? [Single-choice Question] *

○ Never

○ Former smoker

○ Current smoker

Section 2: COVID-19 Vaccination and Infection Status

12. Have you ever received the COVID-19 vaccine? [Single-choice Question] *

○ Yes

○ No

13. How many doses of the COVID-19 vaccine have you received? [Single-choice Question] *

○ 1 dose

○ 2 doses

○ 3 doses

○ 4 doses

(Dependent on the first option selected in Question 12.)

14. Did you experience any of the following reactions within a week after receiving the vaccine? [One or more choice Question] *

□ Local pain and swelling at the injection site

□ Headache

□ Fatigue, drowsiness

□ Muscle pain

□ Diarrhea

□ Cough

□ Fever

□ Abdominal pain

□ No reactions mentioned above

(Dependent on the first option selected in Question 12.)

15. What was the status of your inflammatory bowel disease before COVID-19 infection? [Single-choice Question] *

○ Remission phase

○ Active phase (which requires diagnosis by a third-level hospital physician based on endoscopy, imaging, clinical manifestations, and Chron’s disease activity index, or TrueLove and Witts criteria)

○ Both

○ Not sure

(Dependent on the first option selected in Question 12.)

16. Did you experience any of the following symptoms after receiving the vaccine? [One or more choice Question] *

□ Abdominal pain

□ Diarrhea

□ Nausea

□ Vomiting

□ Increased frequency of bowel movements

□ Rectal bleeding

□ Other _________________*

□ No relevant symptoms

(Dependent on the first option selected in Question 12.)

17. What are the reasons for not receiving the vaccine? [One or more choice Question] *

□ Lack of understanding of the vaccine's efficacy

□ Concerns about the safety of the vaccine

□ Previous strong adverse reactions to vaccination

□ Concerns about potential conflicts between the vaccine and medications for inflammatory bowel disease

□ Concerns about the possibility of vaccine-induced disease relapse

□ Other _________________*

(Dependent on the second option selected in Question 12.)

18. Have you ever been infected by COVID-19? [Single-choice Question] *

○ Yes

○ No

○ Not sure

19. Number of times infected by COVID-19 [Single-choice Question] *

○ Once

○ Twice

○ Three times or more

(Dependent on the first option selected in Question 18.)

20. Date of the most recent COVID-19 infection [Open-ended Question] *

__________________________________

(Dependent on the first option selected in Question 18.)

21. Number of vaccine doses received before the most recent COVID-19 infection [Single-choice Question] *

○ 0 doses

○ 1 dose

○ 2 doses

○ 3 doses

○ 4 doses

(Dependent on the first option selected in Question 18.)

22. Method of confirming the COVID-19 infection [One or more choice Question] *

□ Antigen test

□ Nucleic acid test

(Dependent on the first option selected in Question 18.)

23. How long did it take for your COVID-19 antigen or nucleic acid test to turn negative? [Single-choice Question] *

○ Clear

○ Not clear

(Dependent on the first option selected in Question 18.)

24. How long did it take for your COVID-19 antigen or nucleic acid test to turn negative? (Enter the number of days as a whole number, e.g., 7) [Open-ended Question] *

__________________________________

(Dependent on the first option selected in Question 23.)

Section 3: Medication Usage and Gastrointestinal Symptoms during the Infection

(Dependent on the first option selected in Question 18.)

25. General symptoms during the COVID-19 infection [One or more choice Question] *

□ Fatigue, muscle pain

□ Fever, headache

□ Loss of taste

□ Persistent cough, sore throat

□ Palpitations

□ Abdominal pain

□ Chest pain

□ Rash

□ No obvious symptoms

(Dependent on the first option selected in Question 18.)

26. How many days did it take for the disappearance of the COVID-19-related symptoms mentioned in the previous question? (Enter the number of days as a whole number, e.g., 7) [Open-ended Question] *

__________________________________

(Dependent on the first option selected in Question 18.)

27. Did you seek medical attention for COVID-19 after infection? [Single-choice Question] *

○ Yes

○ No

(Dependent on the first option selected in Question 18.)

28. Did your hospital chest CT indicate pneumonia? [Single-choice Question] *

○ Yes

○ No

○ Chest CT not performed

(Dependent on the first option selected in Question 27.)

29. During the COVID-19 infection, did you have any hospitalization or ICU admission? [Single-choice Question] *

○ Yes

○ No

(Dependent on the first option selected in Question 27.)

30. Inflammatory bowel disease status during the COVID-19 infection [Single-choice Question] *

○ Active phase (which requires diagnosis by a third-level hospital physician based on endoscopy, imaging, clinical manifestations, and Chron’s disease activity index, or TrueLove and Witts criteria)

○ Remission phase

○ Not sure

(Dependent on the first option selected in Question 18.)

31. Are you taking oral medications for controlling inflammatory bowel disease? [Single-choice Question] *

○ Yes

○ No

(Dependent on the first option selected in Question 18.)

32. Types of oral medications used for controlling inflammatory bowel disease [One or more choice Question] *

□ Mesalazine

□ Steroids

□ Azathioprine (Imuran)

□ Methotrexate (MTX)

(Dependent on the first option selected in Question 31.)

33. Have you used biologics for controlling inflammatory bowel disease (such as Infliximab, Adalimumab, Vedolizumab, Ustekinumab, etc.)? [Single-choice Question] *

○ Yes

○ No

(Dependent on the first option selected in Question 18.)

34. Types of biologics used for controlling inflammatory bowel disease [One or more choice Question] *

□ Infliximab (Remicade) / Adalimumab (Humira)

□ Vedolizumab (Entyvio)

□ Interleukin (IL 12/23) inhibitors (Ustekinumab)

(Dependent on the first option selected in Question 33.)

35. Were you in the interval between biologic injections during the COVID-19 infection, where no injection was required? [Single-choice Question] *

○ Yes

○ No

(Dependent on the first option selected in Question 33.)

36. Medication usage for inflammatory bowel disease during the COVID-19 infection (Include the use of biologics if within the injection interval) [One or more choice Question] *

□ Mesalazine

□ Steroids

□ Azathioprine (Imuran)

□ Methotrexate (MTX)

□ Infliximab (Remicade) / Adalimumab (Humira)

□ Vedolizumab (Entyvio)

□ Interleukin (IL 12/23) inhibitors (Ustekinumab)

□ In the interval between biologic injections, no injection needed, biologic used: _________________

(Dependent on the first option selected in Question 18.)

37. Did you discontinue the use of medications for inflammatory bowel disease during the COVID-19 infection? [Single-choice Question] *

○ Yes

○ No

(Dependent on the first option selected in Question 18.)

38. If you discontinued medications for inflammatory bowel disease during the COVID-19 infection, was it under medical advice? [Single-choice Question] *

○ Stopped medication as per medical advice

○ Stopped medication on your own

○ Both situations occurred

(Dependent on the first option selected in Question 37.)

39. Medications stopped during the COVID-19 infection and duration (in weeks) [One or more choice Question] *

□ Mesalazine, duration: _________________*

□ Steroids, duration: _________________*

□ Azathioprine (Imuran), duration: _________________*

□ Methotrexate (MTX), duration: _________________*

□ Infliximab (Remicade) / Adalimumab (Humira), duration: _________________*

□ Vedolizumab (Entyvio), duration: _________________*

□ Interleukin (IL 12/23) inhibitors (Ustekinumab), duration: _________________*

□ Other, medication: _________________*, duration: _________________*

(Dependent on the first option selected in Question 37.)

40. Medications used during COVID-19 [One or more choice Question] *

□ Azithromycin

□ Paxlovid (Nirmatrel/Levothinavir)

□ Corticosteroids

□ Antipyretics (e.g., Tylenol, Ibuprofen)

□ Cough suppressants and expectorants

□ Antibiotics (e.g., cephalosporins, azithromycin, levofloxacin)

□ Traditional Chinese medicine (e.g., Lianhua Qingwen)

□ Other: _________________

□ No medication used

(Dependent on the first option selected in Question 18.)

41. New onset gastrointestinal symptoms during COVID-19 infection [One or more choice Question] *

□ Anorexia

□ Abdominal pain

□ Diarrhea

□ Nausea or vomiting

□ Hematochezia

□ Increased bowel movements

□ Other: _________________

□ No gastrointestinal symptoms

(Dependent on the first option selected in Question 18.)

42. Did you experience persistent gastrointestinal symptoms after a negative result in the COVID-19 antigen or nucleic acid test? [One or more choice Question] *

□ Appetite loss

□ Abdominal pain

□ Diarrhea

□ Nausea

□ Vomiting

□ Rectal bleeding

□ Increased bowel movements

□ Other: _________________

□ No gastrointestinal symptoms

(Dependent on the first option selected in Question 18.)

43. Did you experience weight loss during or after the COVID-19 infection? [Single-choice Question] *

○ Yes

○ No

(Dependent on the first option selected in Question 18.)

44. How much weight did you lose? (Enter the weight loss in kilograms as a whole number, e.g., 5) [Open-ended Question] *

__________________________________

(Dependent on the first option selected in Question 43.)

Section 4: Inflammatory Bowel Disease (IBD) Activity Score in the Week Prior to Infection

Dependent on Question 18, Option 1

45. Times of loose or watery stools in the week prior to infection (Please enter a number, for example: 8) [Fill in the blank] *

_________________________________

Dependent on Question 6, Option 1, and Question 18, Option 1

46. Times and severity of abdominal pain episodes in the week prior to infection (After selecting the severity of abdominal pain, fill in the numbers in the blanks, for example: 8) [Multiple choice, please enter numbers in brackets] *

□ No abdominal pain

□ Mild abdominal pain, number of episodes: ________

□ Moderate abdominal pain, number of episodes: ________

□ Severe abdominal pain, number of episodes: ________

Dependent on Question 6, Option 1, and Question 18, Option 1

47. Overall health status and number of days in the week prior to infection (Total of 7 days, after selecting health status, fill in the numbers in the blank, for example: 2) [Ranking question, please enter numbers in brackets] *

[ ] Good

[ ] Fair

[ ] Poor

[ ] Very poor

[ ] Extremely poor

Dependent on Question 6, Option 1, and Question 18, Option 1

48. Did you experience any of the following conditions in the week prior to infection? [Multiple choice] *

□ Joint pain/arthritis

□ Iritis/uveitis

□ Erythema nodosum/pyoderma gangrenosum/aphthous ulcers

□ Anal fissure/anal fistula/perianal abscess

□ Other fistulas

□ Body temperature >37.8°C in the past week

□ None of the above

Dependent on Question 6, Option 1, and Question 18, Option 1

49. Did you take loperamide/opioid antidiarrheal medication in the week prior to infection? [Single choice] *

○ Yes

○ No

Dependent on Question 6, Option 1, and Question 18, Option 1

50. Did you experience abdominal masses in the week prior to infection? [Single choice] *

○ Yes

○ No

○ Not sure

Dependent on Question 6, Option 1, and Question 18, Option 1

51. Hematocrit (HCT) in the week prior to infection [Selection question, if you are clear, please fill in the decimal number on the line, for example: 0.35] *

○ Clear, Hematocrit value is _________________

○ Not clear

Dependent on Question 6, Option 1, and Question 18, Option 1

52. Number of bloody stools in the week prior to infection (Please enter a number, for example: 7) [Fill in the blank] *

_________________________________

Dependent on Question 6, Option 2, and Question 18, Option 1

53. Did you experience sustained rapid pulse, with a rate exceeding 90 beats per minute in the week prior to infection? [Single choice] *

○ Yes

○ No

Dependent on Question 6, Option 2, and Question 18, Option 1

54. Temperature status in the week prior to infection [Single choice] *

○ No temperature elevation, all below 37.5°C

○ Mild temperature elevation, all below or equal to 37.8°C

○ Temperature elevation above 37.8°C

Dependent on Question 6, Option 2, and Question 18, Option 1

55. Hemoglobin concentration (g/L) in the week prior to infection [Single choice] *

○ All above 115 g/L, no feelings of anemia or fatigue

○ Above 105 g/L, mild feelings of anemia or fatigue

○ Below 105 g/L, significant feelings of anemia or fatigue

Dependent on Question 6, Option 2, and Question 18, Option 1

56. Are you aware of your erythrocyte sedimentation rate (ESR) in the week prior to infection (mm/1h)? [Single choice] *

○ Yes, rate is less than 20 mm/1h

○ Yes, rate is less than or equal to 30 mm/1h, but greater than or equal to 20 mm/1h

○ Yes, rate is greater than 30 mm/1h

○ No, I'm not sure

Dependent on Question 6, Option 2, and Question 18, Option 1

57. Are you aware of your C-reactive protein (CRP) level (mg/L) in the week prior to infection? [Single choice] *

○ Yes, level is normal

○ Yes, level is less than or equal to 30 mg/L

○ Yes, level is greater than 30 mg/L

○ No, I'm not sure

Thank you for your participation in this survey. Wishing you a happy Chinese New Year in 2023 and all the best!
