# Supplementary figures and images for: The association of three vaccination doses with reduced gastrointestinal symptoms after severe acute respiratory syndrome coronavirus 2 infections in patients with inflammatory bowel disease
Source: Front Med (Lausanne). 2024 Mar 18;11:1377926. doi: 10.3389/fmed.2024.1377926 (PMC10982480; doi:10.3389/fmed.2024.1377926)

**Raw Exposed**

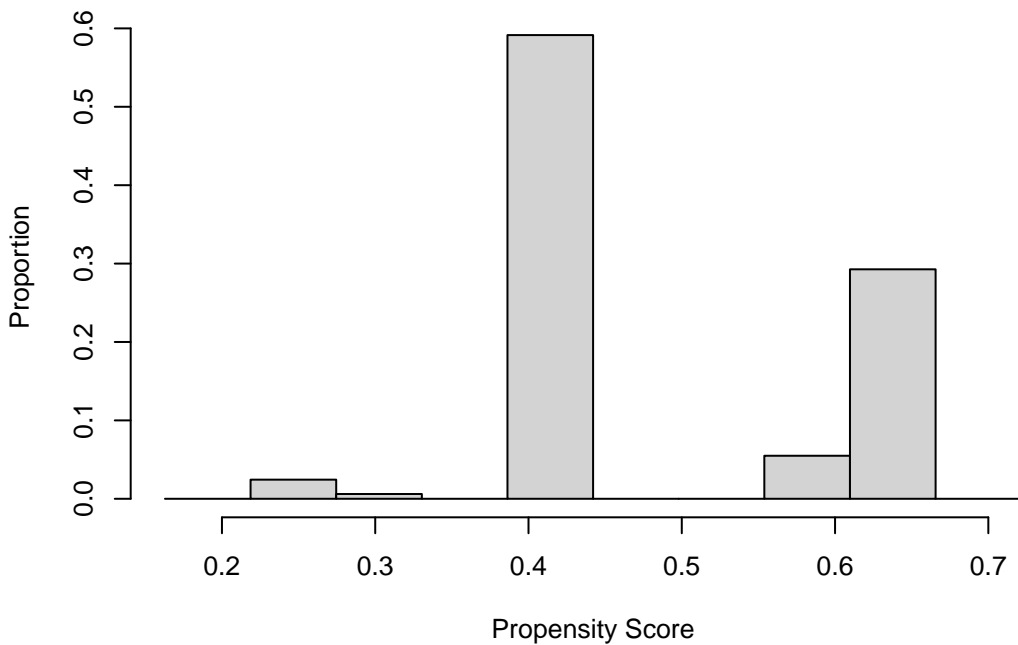

**Matched Exposed**

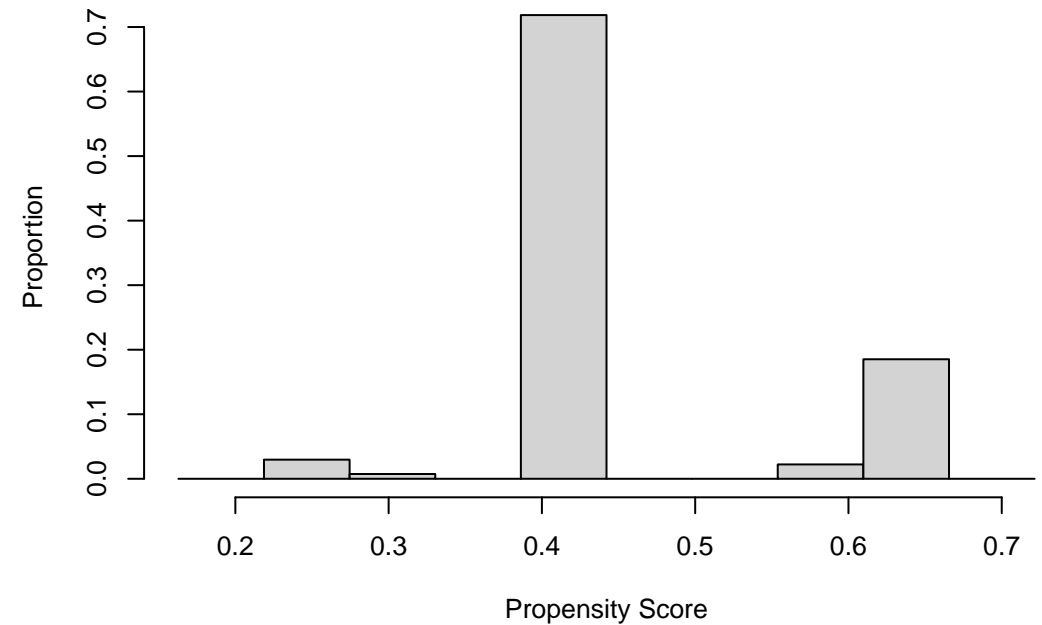

**Raw Nonexposed**

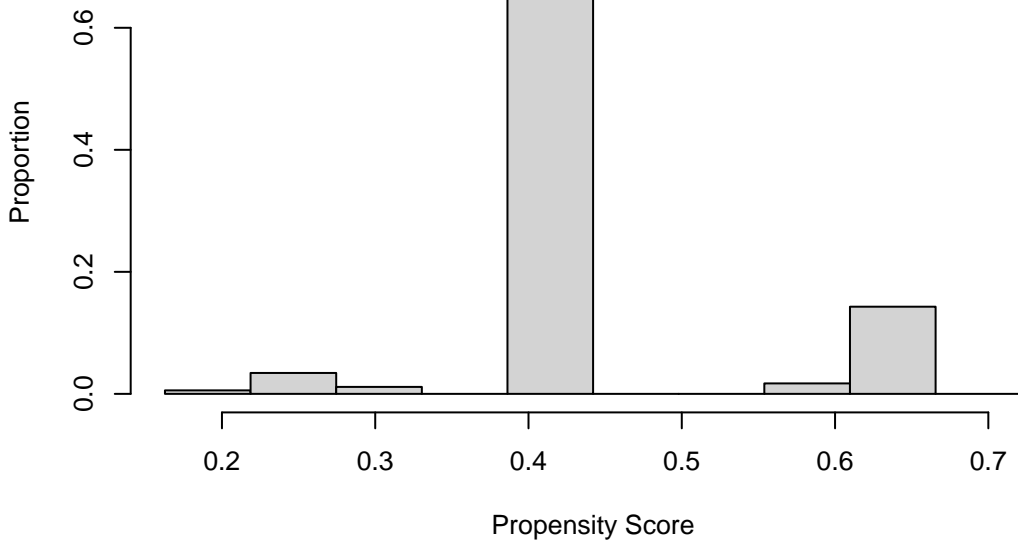

**Matched Nonexposed**

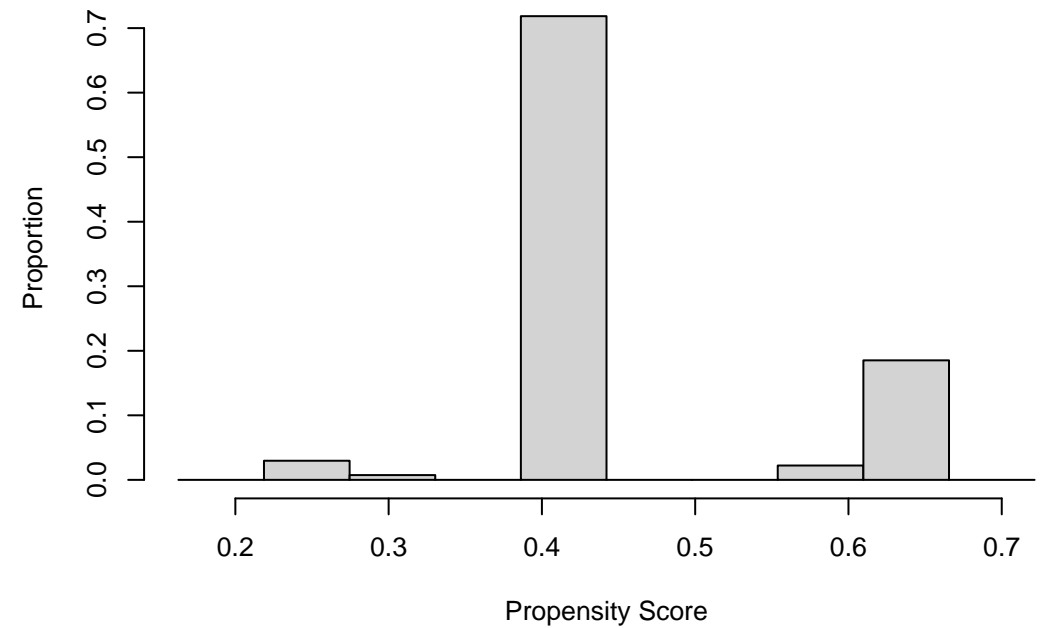

Supplement: Supplementary Figure 1 — Propensity score distribution in the non-exposed and exposed groups. The non-exposed stands for the unvaccinated, the exposed stands for the 3 vaccination doses group. [file Image_1.pdf]

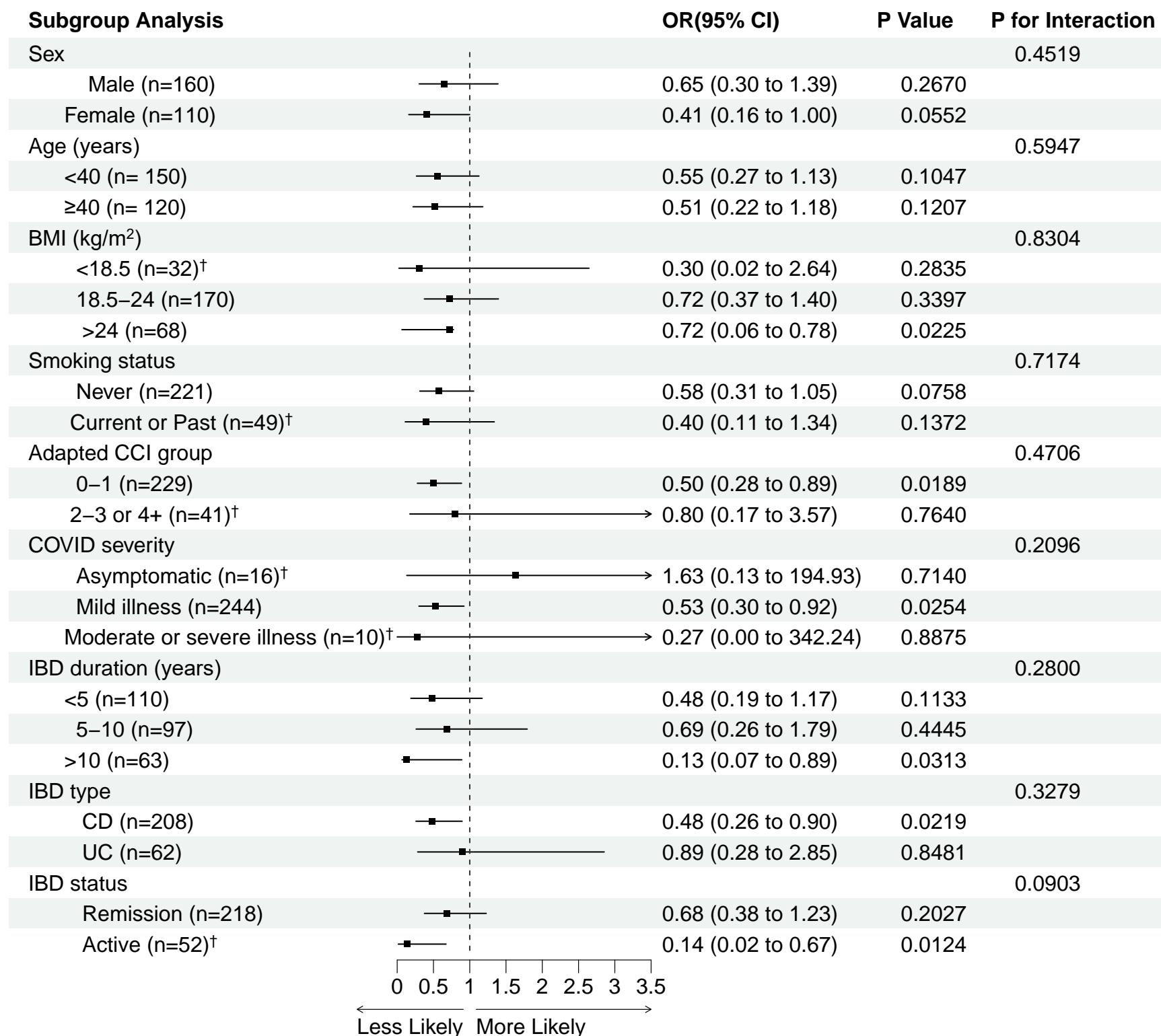

Supplement: Supplementary Figure 2 — Adjusted odds ratios (aORs, 95% confidence intervals) for GI symptomatic infections associated with three vaccination doses in each subgroup vs. the unvaccinated in the matched 270 participants. aORs were calculated using logistic regression models after adjustments for age, sex, BMI, adapted CCI, smoking status, IBD duration, IBD type and IBD status. OR (95% CI), P-values, and P for interaction are calculated in the adjusted model. BMI, body mass index; IBD, inflammatory bowel disease; CD, Crohn's disease; UC, ulcerative colitis; CCI, Charlson comorbidity index.† Logistic regressions with Firth-type penalization were used to analyze these subgroups with case number < 60. [file Image_2.pdf]
