# Supplementary material for: The association of three vaccination doses with reduced gastrointestinal symptoms after severe acute respiratory syndrome coronavirus 2 infections in patients with inflammatory bowel disease
Source: Front Med (Lausanne). 2024 Mar 18;11:1377926. doi: 10.3389/fmed.2024.1377926 (PMC10982480; doi:10.3389/fmed.2024.1377926)
Supplement: Supplementary Table 1 — Characteristics of 116 patients with negative or indetermined infection status. [file Table_1.pdf]

Supplementary Table 1. Characteristics of 116 patients with negative or indetermined infection status

	Unvaccinated (N=41)	1 dose (N=7)	2 doses (N=31)	3 doses (N=37)	P value
Sex					0.072
Male	18 (43.9%)	4 (57.1%)	16 (51.6%)	27 (73.0%)	
Female	23 (56.1%)	3 (42.9%)	15 (48.4%)	10 (27.0%)	
Age (years)	39.0 (15.0%)	34.9 (18.4%)	36.7 (13.0%)	37.2 (13.1%)	0.843
BMI (kg/m^2)	20.7 (3.0%)	19.7 (1.9%)	22.7 (5.0%)	22.7 (4.1%)	0.037
IBD duration (years)					0.713
<5	14 (34.1)	4 (57.1)	15 (48.4)	17 (45.9)	
5-10	17 (41.5)	1 (14.3)	11 (35.5)	14 (37.8)	
>10	10 (24.4)	2 (28.6)	5 (16.1)	6 (16.2)	
IBD type					0.737
CD	32 (78.0%)	6 (85.7%)	22 (71.0%)	26 (70.3%)	
UC	9 (22.0%)	1 (14.3%)	9 (29.0%)	11 (29.7%)	
Smoking status					0.307
Never	34 (82.9%)	7 (100%)	26 (83.9%)	31 (83.8%)	
Past	2 (4.9%)	0 (0%)	5 (16.1%)	3 (8.1%)	
Currrent	5 (12.2%)	0 (0%)	0 (0%)	3 (8.1%)	
Adapted CCI group					0.056
0-1	29 (70.7%)	6 (85.7%)	28 (90.3%)	34 (91.9%)	
2-3	10 (24.4%)	1 (14.3%)	3 (9.7%)	1 (2.7%)	
4+	2 (4.9%)	0 (0%)	0 (0%)	2 (5.4%)	
Infection status					0.198
Uninfected	38 (92.7%)	6 (85.7%)	24 (77.4%)	29 (78.4%)	
Indetermined	3 (7.3%)	1 (14.3%)	7 (22.6%)	8 (21.6%)	

Variables were described using mean (SD) and n (%), as appropriate.

Abbreviations: BMI: body mass index, IBD: inflammatory bowel disease, CD: Crohn's disease, UC: ulcerative colitis, CCI: Charlson comorbidity index, AZA: azathioprine, MTX: methotrexate, TNF: tumor necrosis factor, IL: interleukin.
