# Supplementary material for: The association of three vaccination doses with reduced gastrointestinal symptoms after severe acute respiratory syndrome coronavirus 2 infections in patients with inflammatory bowel disease
Source: Front Med (Lausanne). 2024 Mar 18;11:1377926. doi: 10.3389/fmed.2024.1377926 (PMC10982480; doi:10.3389/fmed.2024.1377926)
Supplement: Supplementary Table 2 — Characteristics of 21 patients excluded in the study with confirmed infection status. [file Table_2.pdf]

Supplementary Table 2. Characteristics of 21 patients excluded in the study with confirmed infection status

	Unvaccinated (N=6)	1 dose (N=1)	2 doses (N=7)	3 doses (N=3)	4 doses (N=4)
Sex					
Male	4 (66.7%)	0 (0%)	6 (85.7%)	1 (33.3%)	4 (100%)
Female	2 (33.3%)	1 (100%)	1 (14.3%)	2 (66.7%)	0 (0%)
Age (years)	41.5 (10.6)	48 (-)	38.9 (14.3)	38.3 (3.8)	31.0 (8.7)
BMI (kg/m^2)	21.6 (3.2)	18 (-)	20.8 (2.1)	20.3 (0.7)	20.4 (4.3)
IBD duration (years)					
<5	3 (50.0%)	1 (100%)	5 (71.4%)	0 (0%)	2 (50%)
5-10	1 (16.7%)	0 (0%)	1 (14.3%)	1 (33.3%)	2 (50%)
>10	2 (33.3%)	0 (0%)	1 (14.3%)	2 (66.7%)	0 (0%)
IBD type					
CD	6 (100%)	0 (0%)	4 (57.1%)	2 (66.7%)	3 (75.0%)
UC	0 (0%)	1 (100%)	3 (42.9%)	1 (33.3%)	1 (25.0%)
Infection times					
1 time	5 (83.3%)	1 (100%)	4 (57.1%)	2 (66.7%)	4 (100%)
2 times	1 (16.7%)	0 (0%)	3 (42.9%)	0 (0%)	0 (0%)
3 times	0 (0%)	0 (0%)	0 (0%)	1 (33.3%)	0 (0%)
Infection time					
Jan-2022	3 (50.0%)	0 (0%)	2 (28.6%)	2 (66.7%)	0 (0%)
Feb-2022	0 (0%)	1 (100%)	0 (0%)	0 (0%)	0 (0%)
Mar-2022	1 (16.7%)	0 (0%)	0 (0%)	0 (0%)	0 (0%)
Apr-2022	1 (16.7%)	0 (0%)	1 (14.3%)	0 (0%)	0 (0%)
May-2022	0 (0%)	0 (0%)	1 (14.3%)	0 (0%)	0 (0%)
Nov-2022	0 (0%)	0 (0%)	1 (14.3%)	0 (0%)	0 (0%)
Dec-2022	1 (16.7%)	0 (0%)	1 (14.3%)	0 (0%)	4 (100%)
Adapted CCI group					
0-1	5 (83.3%)	1 (100%)	6 (85.7%)	3 (100%)	4 (100%)
2-3	1 (16.7%)	0 (0%)	1 (14.3%)	0 (0%)	0 (0%)
COVID_severity					
Asymptomatic	1 (16.7%)	0 (0%)	1 (14.3%)	0 (0%)	0 (0%)
Mild illness	5 (83.3%)	1 (100%)	6 (85.7%)	3 (100%)	4 (100%)

Variables were described using mean (SD) and n (%), as appropriate.

Abbreviations: BMI: body mass index, IBD: inflammatory bowel disease, CD: Crohn's disease, UC: ulcerative colitis, CCI: Charlson comorbidity index.
