# Supplementary material for: The association of three vaccination doses with reduced gastrointestinal symptoms after severe acute respiratory syndrome coronavirus 2 infections in patients with inflammatory bowel disease
Source: Front Med (Lausanne). 2024 Mar 18;11:1377926. doi: 10.3389/fmed.2024.1377926 (PMC10982480; doi:10.3389/fmed.2024.1377926)
Supplement: Supplementary Table 3 — Self-reported adverse events of 152 vaccinated IBD patients using biology agents in the study population. [file Table_3.pdf]

Supplementary Table 3. Self-reported adverse events of 152 vaccinated IBD patients using biology agents in the study population

Adverse Events	1 Dose (N=18)		2 Doses (N=79)		3 Doses (N=55)		p value
	CD (N=18)	UC (N=0)	CD (N=71)	UC (N=8)	CD (N=49)	UC (N=6)	
Local pain or redness (%)	2 (11.1)	-	6 (8.5)	2 (25.0)	8 (16.3)	0 (0.0)	0.021
Headache (%)	1 (5.6)	-	2 (2.8)	0 (0.0)	0 (0.0)	0 (0.0)	0.180
Fatigue (%)	2 (11.1)	-	11 (15.5)	0 (0.0)	4 (8.2)	0 (0.0)	0.016
Muscular pain (%)	1 (5.6)	-	3 (4.2)	1 (12.5)	2 (4.1)	1 (16.7)	0.285
Diarrhea (%)	1 (5.6)	-	4 (5.6)	0 (0.0)	1 (2.0)	0 (0.0)	0.232
Abdominal pain (%)	1 (5.6)	-	4 (5.6)	0 (0.0)	0 (0.0)	0 (0.0)	0.087
Fever (%)	1 (5.6)	-	3 (4.2)	0 (0.0)	1 (2.0)	0 (0.0)	0.338

Variables were described using n (%).

Abbreviations: IBD: inflammatory bowel disease, CD: Crohn's disease, UC: ulcerative colitis.
