# Supplementary material for: The association of three vaccination doses with reduced gastrointestinal symptoms after severe acute respiratory syndrome coronavirus 2 infections in patients with inflammatory bowel disease
Source: Front Med (Lausanne). 2024 Mar 18;11:1377926. doi: 10.3389/fmed.2024.1377926 (PMC10982480; doi:10.3389/fmed.2024.1377926)
Supplement: Supplementary Table 4 — Detailed information of the study population with single anorexia after infection. [file Table_4.pdf]

Supplementary Table 4. Detailed information of the study population with single anorexia after infection

Factor	Unvaccinated (n=15)	1 dose (n=4)	2 doses (n=15)	3 doses (n=21)	P value
Sex					0.051
Male	5 (33.3%)	3 (75.0%)	12 (80.0%)	14 (66.7%)	
Female	10 (66.7%)	1 (25.0%)	3 (20.0%)	7 (33.3%)	
Age	47.7 (18.8)	36.5 (15.3)	31.5 (11.8)	40.9 (13.6)	0.035
BMI	21.5 (2.9)	20.3 (2.5)	20.9 (3.3)	22.9 (4.8)	0.364
IBD duration					0.156
<5	5 (33.3%)	2 (50%)	9 (60.0%)	5 (23.8%)	
5-10	4 (26.7%)	0 (0%)	5 (33.3%)	9 (42.9%)	
>10	6 (40.0%)	2 (50%)	1 (6.7%)	7 (33.3%)	
IBD type					0.396
CD	11 (73.3%)	4 (100%)	13 (86.7%)	14 (66.7%)	
UC	4 (26.7%)	0 (0%)	2 (13.3%)	7 (33.3%)	
Smoking status					0.477
Never	13 (86.7%)	4 (100%)	10 (66.7%)	18 (85.7%)	
Past	2 (13.3%)	0 (0%)	4 (26.7%)	3 (14.3%)	
Current	0 (0%)	0 (0%)	1 (6.7%)	0 (0%)	
Adapted CCI					0.026
0-1	11 (73.3%)	4 (100%)	15 (100%)	16 (76.2%)	
2-3	1 (6.7%)	0 (0%)	0 (0%)	5 (23.8%)	
4+	3 (20.0%)	0 (0%)	0 (0%)	0 (0%)	
IBD status					0.396
Remission	12 (80.0%)	4 (100%)	12 (80.0%)	20 (95.2%)	
Active	3 (20.0%)	0 (0%)	3 (20.0%)	1 (4.8%)	
COVID severity					
Asymptomatic	1 (6.7%)	0 (0%)	0 (0%)	0 (0%)	
Mild illness	13 (86.7%)	4 (100%)	15 (100%)	19 (90.5%)	
Moderate illness	1 (6.7%)	0 (0%)	0 (0%)	1 (4.8%)	
Severe illness	0 (0%)	0 (0%)	0 (0%)	1 (4.8%)	
IBD Medication Group					
5-ASA	4 (26.7%)	0 (0%)	4 (26.7%)	3 (14.3%)	0.615
Corticosteroid	0 (0%)	0 (0%)	1 (6.7%)	0 (0%)	0.618
MTX/AZA	0 (0%)	0 (0%)	2 (13.3%)	0 (0%)	0.283
Anti-TNF	4 (26.7%)	2 (50.0%)	8 (53.3%)	4 (19.0%)	0.138
Anti-α4β7-integrins	2 (13.3%)	0 (0%)	0 (0%)	1 (4.8%)	0.580
Anti-IL-12/IL-23	2 (13.3%)	1 (25.0%)	2 (13.3%)	0 (0%)	0.268

Variables were described using mean (SD) and n (%), as appropriate.

Abbreviations: IBD: inflammatory bowel disease, CD: Crohn's disease, UC: ulcerative colitis, CCI: Charlson comorbidity index, AZA: azathioprine, MTX: methotrexate, TNF: tumor necrosis factor, IL: interleukin.
