# Supplementary material for: The association of three vaccination doses with reduced gastrointestinal symptoms after severe acute respiratory syndrome coronavirus 2 infections in patients with inflammatory bowel disease
Source: Front Med (Lausanne). 2024 Mar 18;11:1377926. doi: 10.3389/fmed.2024.1377926 (PMC10982480; doi:10.3389/fmed.2024.1377926)
Supplement: Supplementary Table 5 — Score table of adapted Charlson comorbidity index in the study. [file Table_5.pdf]

Supplementary Table 5. Score table of adapted Charlson comorbidity index in the study

Item	Score
Age	
<50	0
50-59	1
60-69	2
70-79	3
≥80	4
Comorbidity disease	
Cardiovascular Disease	1
Cerebrovascular Disease	1
Chronic lung disease	1
Chronic liver disease	1
Kidney disease	1
Diabetes	1
Tumor	2
Other disease	1
