# Supplementary material for: The association of three vaccination doses with reduced gastrointestinal symptoms after severe acute respiratory syndrome coronavirus 2 infections in patients with inflammatory bowel disease
Source: Front Med (Lausanne). 2024 Mar 18;11:1377926. doi: 10.3389/fmed.2024.1377926 (PMC10982480; doi:10.3389/fmed.2024.1377926)
Supplement: Supplementary Table 6 — Detailed comorbidity information of the study population. [file Table_6.pdf]

Supplementary Table 6. Detailed comorbidity information of the study population

	Unvaccinated (N=175)	1 Dose (N=31)	2 Doses (N=166)	3 Doses (N=164)	P value
Age (years)					0.129
<50	131 (74.9%)	23 (74.2%)	147 (88.6%)	120 (73.2%)	
50-59	24 (13.7%)	4 (12.9%)	9 (5.4%)	24 (14.6%)	
60-69	13 (7.4%)	2 (6.5%)	9 (5.4%)	15 (9.1%)	
70-79	6 (3.4%)	2 (6.5%)	0 (0%)	5 (3.0%)	
⩾80	1 (0.6%)	0 (0%)	1 (0.6%)	0 (0%)	
Comorbidity disease					
No conditions	145 (82.9%)	22 (71.0%)	142 (85.5%)	127 (77.4%)	0.110
Cardiovascular disease	15 (8.6%)	6 (19.4%)	9 (5.4%)	16 (9.8%)	0.070
Cerebrovascular disease	1 (0.6%)	0 (0%)	1 (0.6%)	2 (1.2%)	0.763
Chronic lung disease	4 (2.3%)	1 (3.2%)	3 (1.8%)	9 (5.5%)	0.217
Chronic liver disease	1 (0.6%)	1 (3.2%)	4 (2.4%)	9 (5.5%)	0.046
Kidney disease	5 (2.9%)	2 (6.5%)	0 (0%)	1 (0.6%)	0.016
Diabetes	3 (1.7%)	1 (3.2%)	0 (0%)	3 (1.8%)	0.294
Tumor	6 (3.4%)	1 (3.2%)	2 (1.2%)	1 (0.6%)	0.218
Other diseases	6 (3.4%)	1 (3.2%)	7 (4.2%)	5 (3.0%)	0.951

Variables were described using n (%).
