# Supplementary material for: The association of three vaccination doses with reduced gastrointestinal symptoms after severe acute respiratory syndrome coronavirus 2 infections in patients with inflammatory bowel disease
Source: Front Med (Lausanne). 2024 Mar 18;11:1377926. doi: 10.3389/fmed.2024.1377926 (PMC10982480; doi:10.3389/fmed.2024.1377926)
Supplement: Supplementary Table 7 — Baseline characteristics of participants of unvaccinated and 3 doses vaccination after propensity score matching. [file Table_7.pdf]

Supplementary Table 7. Baseline characteristics of participants of unvaccinated and 3 doses vaccination after propensity score matching.

Factor	Unvaccinated (N=135)	3 doses (N=135)	P value
Sex			0.001
Male	66 (48.9)	94 (69.6)	
Female	69 (51.1)	41 (30.4)	
Age (years)	40.04 (13.80)	39.17 (13.99)	0.609
BMI (kg/m^2)	21.92 (3.09)	22.14 (3.84)	0.592
Smoking status			
Never	115 (85.2)	106 (78.5)	0.364
Past	16 (11.9)	23 (17.0)	
Current	4 (3.0)	6 (4.4)	
CCI group			
0-1	115 (85.2)	114 (84.4)	0.894
2-3	12 (8.9)	14 (10.4)	
4+	8 (5.9)	7 (5.2)	
COVID severity			1
Asymptomatic	8 (5.9)	8 (5.9)	
Mild illness	122 (90.4)	122 (90.4)	
Moderate illness	4 (3.0)	4 (3.0)	
Severe illness	1 (0.7)	1 (0.7)	
IBD duration (years)			0.017
<5	47 (34.8)	63 (46.7)	
5-10	47 (34.8)	50 (37.0)	
>10	41 (30.4)	22 (16.3)	
IBD type			
CD	104 (77.0)	104 (77.0)	1
UC	31 (23.0)	31 (23.0)	
IBD status			
Remission	106 (78.5)	112 (83.0)	0.548
Active	29 (21.5)	23 (17.0)	
IBD medication			
5-ASA	24 (17.8)	30 (22.2)	0.447
Corticosteroid	3 (2.2)	5 (3.7)	0.720
MTX/ AZA	22 (16.3)	13 (9.6)	0.147
Anti-TNF	35 (25.9)	30 (22.2)	0.569
Anti-α4β7-integrins	13 (9.6)	5 (3.7)	0.088
Anti-IL-12/IL-23	12 (8.9)	12 (8.9)	1

Variables were described using mean (SD) and n (%), as appropriate.

Abbreviations: BMI: body mass index, IBD: inflammatory bowel disease, CD: Crohn's disease, UC: ulcerative colitis, CCI: Charlson comorbidity index, AZA: azathioprine, MTX: methotrexate, TNF: tumor necrosis factor, IL: interleukin.
