# Supplementary material for: The association of three vaccination doses with reduced gastrointestinal symptoms after severe acute respiratory syndrome coronavirus 2 infections in patients with inflammatory bowel disease
Source: Front Med (Lausanne). 2024 Mar 18;11:1377926. doi: 10.3389/fmed.2024.1377926 (PMC10982480; doi:10.3389/fmed.2024.1377926)
Supplement: Supplementary Table 8 — Association of vaccination doses and the occurrence of GI symptoms among 270 patients after propensity score matching. [file Table_8.pdf]

Supplementary Table 8. Association of vaccination dosages and the occurrence of GI symptoms among 270 patients after propensity score matching.

Group	Vaccination status	GI symptomatic positive /negative cases	OR (95% CI)		P value [a]
			Unadjusted	Adjusted [c]	
IBD (n=270)	Unvaccinated	72/63	Ref	Ref	Ref
	3 Doses	52/83	0.55 (0.34-0.89)	0.53 (0.30-0.93)	0.027
CD (n=208)	Unvaccinated	57/47	Ref	Ref	Ref
	3 Doses	40/64	0.52 (0.30-0.89)	0.48 (0.25-0.90)	0.025
UC (n=62)	Unvaccinated	15/16	Ref	Ref	Ref
	3 Doses	12/19	0.67 (0.24-1.84)	1.44 (0.29-7.75)	0.657

Abbreviation: OR, odds ratio; GI symptom, gastrointestinal symptom.

[a] P value for the comparison of the adjusted OR for 3 doses vs unvaccinated.

[b] An adjusted OR less than 1 indicated that receipt of 3 doses (vs unvaccinated) was less likely among GI symptom positive cases vs GI symptom negative cases. Models included age, sex, BMI, adapted CCI (0-1, 2-3, 4+), smoking status, IBD duration, IBD type, IBD status, IBD medications (see **Supplementary Table 7**).
